# Comparison of Two Techniques of Superior Vena Cava Flow Measurement in Preterm Infants With Birth Weight <1,250 g in the Transitional Period—Prospective Observational Cohort Study

**DOI:** 10.3389/fped.2021.661698

**Published:** 2021-04-07

**Authors:** Jan Miletin, Zbynek Stranak, Niamh Ó Catháin, Jan Janota, Jana Semberova

**Affiliations:** ^1^Coombe Women and Infants University Hospital, Dublin, Ireland; ^2^Institute for the Care of Mother and Child, Prague, Czechia; ^3^UCD School of Medicine, University College Dublin, Dublin, Ireland; ^4^3rd Faculty of Medicine, Charles University, Prague, Czechia; ^5^1st and 2nd Faculty of Medicine, Charles University, Prague, Czechia; ^6^Motol University Hospital, Prague, Czechia

**Keywords:** superior vena cava, flow, cardiac output, preterm neonate, technique

## Abstract

**Objectives:** Superior Vena Cava (SVC) flow in neonates measured by the standard approach has been validated by different groups around the world. The modified SVC flow measurement technique was recently suggested. The aim of our study was to evaluate standard and modified technique of echocardiography SVC flow measurement in a cohort of extremely preterm neonates in the immediate postnatal period.

**Methods:** Prospective, observational cohort study in a level III neonatal center. Infants with birth weight <1,250 g were eligible for enrolment. SVC flow was measured by echocardiography using standard and modified methods at 6, 18 and 36 h of age. Our primary outcome was equivalency (using raw bounds of −20 to +20 mL/kg/min difference between the paired measurements), agreement and correlation between standard and modified methods of the SVC flow measurements.

**Results:** Thirty-nine infants were enrolled. The mean gestational age of the cohort was 27.4 (SD 2.1) weeks of postmenstrual age, the mean birth weight was 0.95 kg (SD 0.2). The measurements at 6 and 36 h of age were equivalent as defined in the design of the study (*p* = 0.003 and *p* = 0.004 respectively; raw bounds −20 to +20 mL/kg/min). At 6 h of age the mean difference (bias) between the measurements was −0.8 mL/kg/min with 95% limits of agreement −65.0 to 63.4 mL/kg/min. At 18 h of age, the mean difference (bias) between the measurements was +9.5 mL/kg/min, with 95% limits of agreement −79.6 to 98.7 mL/kg/min. At 36 h of age the mean difference (bias) between the measurements was −2.2 mL/kg/min with 95% limits of agreement −73.4 to 69.1 mL/kg/min. There was a weak, but statistically significant correlation between the standard and modified method at 6 h of age (*r* = 0.39, *p* = 0.04).

**Conclusion:** Both SVC flow echocardiography measurement techniques yielded clinically equivalent results, however due to wide limits of agreement and poor correlation they do not seem to be interchangeable.

## Introduction

Invasive measurement of cardiac output, currently employed in adult and pediatric intensive care medicine, is not technically feasible in preterm infants due to lack of appropriately sized devices (1). Therefore, the cardiac output measured by echocardiography, together with the other echocardiography markers of cardiac function, has gained a lot of attention amongst neonatal practitioners over the last 20 years (2). Such measurement is technically feasible, non-invasive, and well-tolerated, but in the newborn period and even more in premature infants it is quite imprecise as a result of naturally occurring shunts between the systemic and pulmonary circulation—patent ductus arteriosus and foramen ovale. The effect of the ductal shunt on left ventricular output, and of atrial shunts on right ventricular output, can cause either of these measurements to overestimate the real systemic blood flow by up to 100% (3–5).

To overcome this issue, a Superior Vena Cava (SVC) flow measurement technique has been developed in preterm infants. SVC flow is independent of the shunts and 80% of SVC flow consists of blood return from cerebral circulation and can be a surrogate marker of systemic blood flow (5). Low SVC flow, measured by the standard approach described by Kluckow et al., has been associated with increased risk of intraventricular hemorrhage (IVH) and adverse neurodevelopmental outcomes. Low SVC flow is related to reduced urinary output and subsequent hyperkalaemia in preterm infants (6–9). A relationship between amplitude-integrated electroencephalography and SVC flow has been reported (10). The technique became a standard approach to SVC flow measurement and was validated by different groups around the world (6, 7, 11–18). Furthermore this technique would be considered standard approach to SVC flow measurement by expert consensus (19). The velocity time integral (VTI) is measured from a subxiphoid trace, and a parasternal view in a true sagittal plane is used to measure the diameter of the SVC using 2D or M-mode imaging and then the SVC area is calculated (5, 20). The standard SVC flow echocardiography assessment technique has been somewhat challenging and has high intra-observer variability (median ranging between 8 and 12%) and inter-observer variability (median 14–56%) in different studies (5, 11, 21). However, with highly experienced operators, the median inter-observer variability was as low as 6% (22, 23).

Ficial et al. demonstrated poor correlation between standard SVC flow measurement and MRI SVC flow assessment. The modified SVC flow measurement technique was suggested by the same group, using measurement of SVC area from an axial view and applying 50% reduction to stroke distance to compensate for overestimation by the subxiphoid approach (24). Subsequently, the modified approach included VTI measured from the suprasternal view (25). This modified measurement offered an improvement in accuracy and repeatability in a study by Ficial et al. (26).

However, both reports using modified approach of SVC flow were done mostly on infants beyond 48 h of age and on infants with gestation varying from extreme prematurity to term gestation.

The aim of our study was to evaluate the standard and modified techniques of echocardiography SVC flow measurement in a cohort of extremely preterm infants in the immediate postnatal period. We aimed to describe equivalency, correlation and agreement between the two techniques, and variability of components of the calculations in infants born below 1,250 g at 6, 18, and 36 h of age.

## Materials and Methods

This was a prospective, single center, observational study in Coombe Women and Infants University Hospital (CWIUH), Dublin, Ireland (level III perinatal center). The enrolment period was planned for 18 months (January 2016 to June 2017). The study was approved by the Research Ethics Committee in CWIUH (No.23-2015).

### Participants

Inclusion criteria for the study were: birth weight <1,250 g, age <36 h at the time of enrolment, baseline cranial ultrasound free of IVH ≥grade II (performed before first echocardiography), parental consent and presence of a researcher capable of SVC flow measurements (JM, JS). Infants with major congenital and/or chromosomal anomalies (including congenital heart diseases other than patent ductus arteriosus or foramen ovale) were excluded from participation, as were infants where the decision by the attending physician was to provide palliative and/or comfort care only.

### Measurements

All infants were planned to have a cranial ultrasound just prior to the first echocardiography assessment to rule out any intracranial abnormality and/or IVH. The normal anatomy of the heart was established at the first echocardiography. Infants enrolled in the study had standard and modified SVC flow measurements at 6, 18, and 36 h of age. We allowed for infants to be enrolled to the study at any time before 36 h of age to have at least one paired measurement, however we aimed for enrolment before 6 h of age where possible. All echocardiography measurements were done by one of two researchers (JM, JS) trained in both methods of SVC flow measurements. All paired studies were done by the same investigator.

#### Echocardiography

Evaluations were performed by Phillips CX50 (Philips, Amsterdam, Netherlands) echocardiography system with the sector 12 Hz cardiology probe. All studies were archived and reviewed later to assess quality and accuracy of data acquisition. All measurements were done off-line after finishing the study. Standard views were obtained at the first echocardiography to confirm normal heart anatomy (19).

#### SVC Flow Measurement–Standard Method (5)

SVC diameter was assessed using parasternal long axis view with the beam in a true sagittal plane and angled to the right of the ascending aorta. M–mode was used for measurements of minimal and maximal diameter during the cardiac cycle. Diameters were measured at the point where the SVC starts to open up into the right atrium. Diameter measurements were averaged from three cardiac cycles and the sweep setting of the machine was 100 mm/s. Diameter was measured in centimeters (cm) for further calculations and was averaged from all six measurements (three minimal and three maximal diameters).

Velocity Time Integral (VTI) was measured using low subcostal view. The SVC flow was identified by angling the beam anteriorly and by using color Doppler. The angle of insonation was minimized by maneuvering the transducer inferiorly, without software corrections. The SVC flow was measured by pulsed wave (PW) Doppler at the junction of the SVC and the right atrium. A representative sample of at least 20 cycles was obtained at the pre-set sweep of 100 mm/s. The mean velocity of blood flow was calculated from the integral of the Doppler velocity tracings and was averaged from five consecutive cardiac cycles. SVC VTI was expressed in cm. Forward flow was positively integrated and any retrograde flow was negatively integrated.

The heart rate was recorded by the ECG leads. If this was not possible, images obtained for SVC VTI were used and the heart rate was measured from the intervals between the cardiac cycles. Birth weight of the patient was used for all calculations.

Calculation of the SVC flow = (SVC VTI × (π × (mean SVC diameter^2^/4) × heart rate)/body weight. The resulting figure has been expressed in mL/kg/min.

#### SVC Flow Measurement–Modified Method (24, 25)

SVC area was assessed directly from the axial/short axis view. B-mode images were obtained, and we traced maximum and minimum cross-sectional SVC area in three consecutive heart cycles. SVC area was then averaged from all measurements, expressed in cm^2^.

VTI was measured from the high midline/up to suprasternal or parasternal view as needed, to imagine the SVC as close as possible to its junction with the right atrium and distal from the azygos confluence. The aim was to ensure the smallest possible angle between the Doppler beam and the vessel axis. The SVC flow was visualized with color Doppler imaging and measured by PW Doppler. A representative sample of at least 20 cycles was obtained at the pre-set sweep of 100 mm/s. The mean velocity of blood flow was calculated from the integral of the Doppler velocity tracings and was averaged from five consecutive cardiac cycles. SVC VTI was expressed in cm. Any forward flow was positively integrated and any retrograde flow was negatively integrated. The angle correction was allowed when the angle between the SVC at the point of measure and the ultrasound beam was >15°, but better avoided.

The heart rate was recorded by the ECG leads. In case this was not possible, images obtained for SVC VTI were used and the heart rate was measured from the intervals between the cardiac cycles. Birth weight of the patient was used for all calculations.

Calculation of the SVC flow = (SVC VTI × SVC area × heart rate)/body weight. The resulting figure was expressed in mL/kg/min.

#### Cranial Ultrasound

Bedside cranial ultrasound was performed by an investigator trained to perform this procedure at the time of enrolment. The aim of the ultrasound was only to rule out any major intracranial abnormality including IVH ≥grade II (27). Phillips CX50 (Philips, Amsterdam, Netherlands) ultrasound system with a curved 8 Hz probe was used.

Results of the SVC flow measurements were recorded after 36 h of age on pre-specified pro-forma sheet together with basic demographic parameters: gestational age, birth weight, Apgar scores, gender and timings of all SVC flow measurements in the study (planned for 6, 18, and 36 h of age).

Pro-forma sheets were collected in the study folder in neonatal intensive care unit (NICU) and then pseudo-anonymised and stored electronically in Excel sheet (Microsoft Excel, USA) for the statistical analysis in the password protected computer.

### Outcomes

Our primary outcome was equivalency, correlation and agreement between standard and modified method of the SVC flow measurement. Our secondary outcome was correlation and agreement between components of the SVC flow calculation, namely SVC VTI, SVC cross-sectional area and heart rate.

### Statistics

All demographic parameters were expressed using mean (SD), median (IQR) or percentages as appropriate. We have defined SVC flow equivalency using raw bounds of −20 to +20 mL/kg/min difference between the paired measurements and used two-one-sided *t*-test (TOST) equivalency test with 90% Confidence Intervals. Correlation between the standard and modified methods and their components was calculated using Pearson's correlation coefficient. Bland-Altman (B-A) analysis was used to calculate and visualize the agreement between the standard and modified SVC flow measurement and their components. The agreement limits are demonstrated as a 95% confidence interval (95% CI = mean ± 1.96 standard deviations), where the ideal agreement difference between measurements is zero. Paired-samples *t*-test was used to compare paired measurements and their components. When the differences between pairs were not normally distributed, we used the Wilcoxon signed-rank test for two sample comparisons. *P*-values <0.05 were considered statistically significant.

The data were analyzed by PC-based statistics software, StatsDirect version 3.2.10 (StatsDirect Ltd, United Kingdom). For equivalency testing, R software (R 4.0.0, The R Foundation, https://www.r-project.org/) was used.

## Results

We enrolled 39 infants between January 2016 and July 2017 who fulfilled inclusion criteria and had at least one pair of SVC flow measurements (at 6, 18, and/or 36 h of age). The mean gestational age of the cohort was 27.4 (SD 2.1) weeks of postmenstrual age, the mean birth weight was 0.95 kg (SD 0.2). The median Apgar score at 1st min was 7 (IQR 5, 9) and median Apgar score at 5th min was 9 (IQR 8, 9). There were 23 (59%) females in the cohort. The respiratory and inotropic support at each time point is presented in Table 1. The mean time (SD) of the measurements was 7.8 (1.3) h for 6 h of age, 19.3 (1.8) h for 18 h of age and 37.7 (2.7) h for 36 h of age.

**Table 1:** Respiratory and inotropic support for infants enrolled to the study.

***n* = 39**	**6 h of age**	**18 h of age**	**36 h of age**
No support, *n* (%)	0 (0)	0 (0)	2 (5)
nCPAP, *n* (%)	28 (72)	32 (82)	33 (85)
Conventional Ventilation, *n* (%)	11 (28)	7 (18)	4 (10)
HFOV, *n* (%)	0 (0)	0 (0)	0 (0)
FiO_2_, mean (SD)	0.25 (0.06)	0.25 (0.1)	0.24 (0.06)
Inotropic support, *n* (%)	1 (3)	1 (3)	1 (3)

*nCPAP, nasal Continuous Positive Airway Pressure; HFOV, High Frequency Oscillatory Ventilation; FiO_2_, Fraction of Inspired Oxygen*.

The number of paired measurements for the standard and modified approach of SVC flow were 27 at 6 h of age, 32 at 18 h of age and 33 at 36 h of age.

The measurements at 6 and 36 h of age were equivalent as defined in the design of the study (raw bounds −20 to +20 mL/kg/min). The SVC flow at three different time points as measured by standard and modified method is presented in Table 2.

**Table 2:** Superior Vena Cava (SVC) flow at three different time points and TOST (two-one-sided *t*-tests) equivalency test (raw bounds −20 to +20 mL/kg/min).

	**Standard Method Mean (SD); Median (IQR)**	**Standard Method Mean (SD); Median (IQR)**	**TOST Equivalency *p* (for raw bounds −20 to +20 mL/kg/min (90% Confidence IntervalmL/kg/min)**
SVC 6 h of age (mL/kg/min)	76.8 (27.7); 67.9 (57.8, 91.2)	77.6 (31.5); 74.5 (57.9, 85.3)	0.003 (−11.6 to 10.0)
SVC 18 h of age (mL/kg/min)	92.3 (34.9); 79.5 (68.0, 114.7)	82.8 (32.4); 77.4 (60.5, 91.3)	0.1 (−4.1 to 23.1)
SVC 36 h of age (mL/kg/min)	82.2 (27.1); 80.6 (60.2, 92.9)	84.3 (29.3); 83.3 (60.8, 101.5)	0.004 (−12.8 to 8.6)

**Figure 1:**
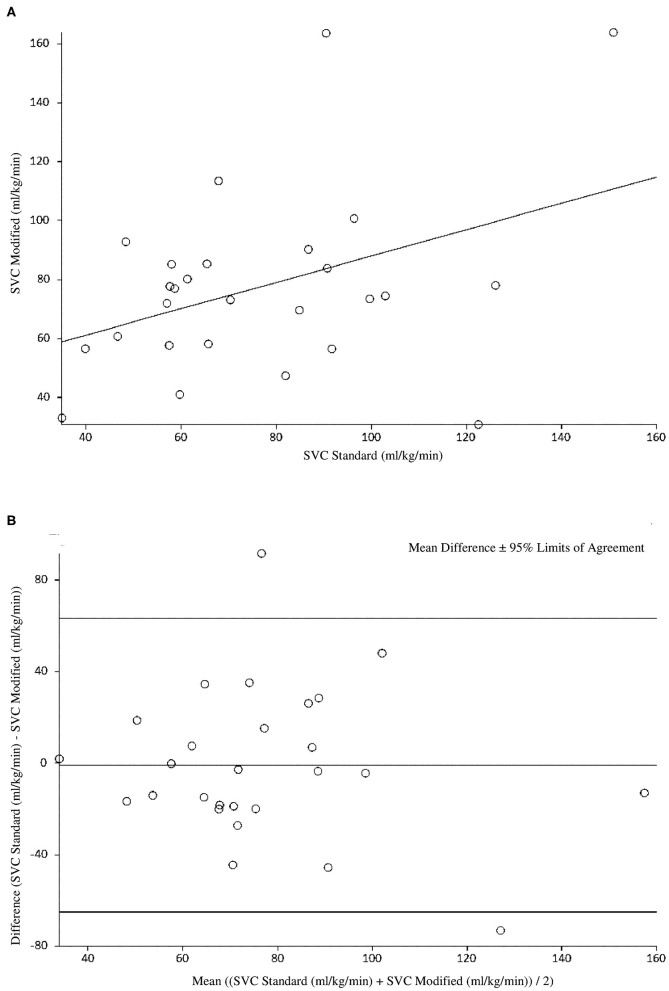
**(A)** Correlation between standard and modified Superior Vena Cava (SVC) flow measurement at 6 h of age. **(B)** Agreement between standard and modified Superior Vena Cava (SVC) Flow measurement at 6 h of age with mean difference and 95% limits of agreement.

At 6 h of age, the standard SVC flow method had statistically significant correlation with the modified method, however this correlation was weak (*r* = 0.39, *p* = 0.04). The mean difference (bias) between the measurements was −0.8 mL/kg/min with 95% limits of agreement −65 to 63.4 mL/kg/min (Figures 1A,B, Tables 3, 4). The heart rate correlated strongly and significantly between the two methods at this time point. Area of the SVC correlated significantly between the methods. There was also weak, but statistically significant correlation for a VTI at 6 h of age between both methods (Table 3). The mean of differences, median of differences and 95% limits of agreement for the area of the SVC, VTI and heart rate for 6 h of age are included in Table 4.

**Table 3:** Correlation coefficients for standard Superior Vena Cava (SVC) flow measurement method and modified method, including all measurements used for the calculation of SVC flow for three time points.

	**Standard Method Mean (SD)**	**Modified Method Mean (SD)**	***r* (*r*^**2**^)**	***p***
**6 h of age**, ***n*** **=** **27**				
SVC flow (mL/kg/min)	76.8 (27.7)	77.6 (31.5)	0.39 (0.15)	0.04
- Heart Rate (beats/min)	153 (14)	151 (14)	0.72 (0.52)	<0.0001
- Velocity Time Integral (cm)	9.2 (2.9)	6.1 (1.8)	0.54 (0.29)	0.003
- SVC area (cm^2^)	0.05 (0.01)	0.08 (0.03)	0.70 (0.49)	<0.0001
**18 h of age**, ***n*** **=** **32**				
SVC flow (mL/kg/min)	92.3 (34.9)	82.8 (32.4)	0.09 (0.008)	0.64
- Heart Rate (beats/min)	150 (13)	148 (12.7)	0.75 (0.56)	<0.0001
- Velocity Time Integral (cm)	10.0 (2.6)	7.0 (2.7)	0.28 (0.08)	0.12
- SVC area (cm^2^)	0.06 (0.02)	0.08 (0.02)	0.53 (0.28)	0.002
**36 h of age**, ***n*** **=** **33**				
SVC flow (mL/kg/min)	82.2 (27.1)	84.3 (29.3)	0.17 (0.03)	0.35
- Heart Rate (beats/min)	154 (15)	151 (13)	0.70 (0.49)	<0.0001
- Velocity Time Integral (cm)	9.8 (2.8)	6.7 (2.2)	0.24 (0.06)	0.17
- SVC area (cm^2^)	0.05 (0.02)	0.08 (0.02)	0.30 (0.09)	0.09

**Table 4:** Mean and median difference (bias) between the standard and modified method of superior vena cava (SVC) flow measurement with *p*-values (paired-t or Wilcoxon's ranked signed rank test as appropriate) and 95% limits of agreement, including cross sectional area of SVC, Velocity Time Integral (VTI) and heart rate at all three time points.

	**Mean of Differences (SD) Median of Differences (IQR)**	***p***	**95% Limits of Agreement**
**6 h of age**			
SVC Flow Standard vs. SVC Flow Modified method (mL/kg/min)	−0.80 (32.7); −3.46 (−18.6, 17.0)	0.90	−65.0 to 63.4
VTI Standard vs. VTI Modified method (cm)	3.1 (2.4); 2.5 (1.5, 4.0)	<0.0001	−1.7 to 7.9
SVC area Standard vs. Modified method (cm^2^)	−0.029 (0.020); −0.031 (−0.038, −0.022)	<0.0001	−0.068 to 0.009
SVC heart rate Standard vs. Modified method (beats/min)	1.9 (10.5); 0 (−3.5, 9)	0.35	−18.6 to 22.5
**18 h of age**			
SVC Flow Standard vs. SVC Flow Modified method (mL/kg/min)	9.52 (45.5); 9.48 (−16.4, 28.2)	0.25	−79.6 to 98.7
VTI Standard vs. VTI Modified method (cm)	3.0 (3.2); 3.1 (0.5, 4.6)	<0.0001	−3.2 to 9.2
SVC area Standard vs. Modified method (cm^2^)	−0.018 (0.021); −0.014 (−0.031, −0.005)	<0.0001	−0.060 to 0.023
SVC heart rate Standard vs. Modified method (beats/min)	1.7 (8.9); 2.5 (−3, 8)	0.30	−15.8 to 19.1
**36 h of age**			
SVC Flow Standard vs. SVC Flow Modified method (mL/kg/min)	−2.15 (36.4); −0.64 (−30.1, 12.8)	0.46	−73.4 to 69.1
VTI Standard vs. VTI Modified method (cm)	3.1 (3.1); 2.8 (1.4, 4.5)	<0.0001	−3.0 to 9.3
SVC Area Standard vs. Modified method (cm^2^)	−0.028 (0.022); −0.033 (−0.040, −0.019)	<0.0001	−0.072 to 0.016
SVC heart rate Standard vs. Modified method (beats/min)	2.5 (10.8); 3 (−4, 7)	0.18	−18.6 to 23.6

**Figure 2:**
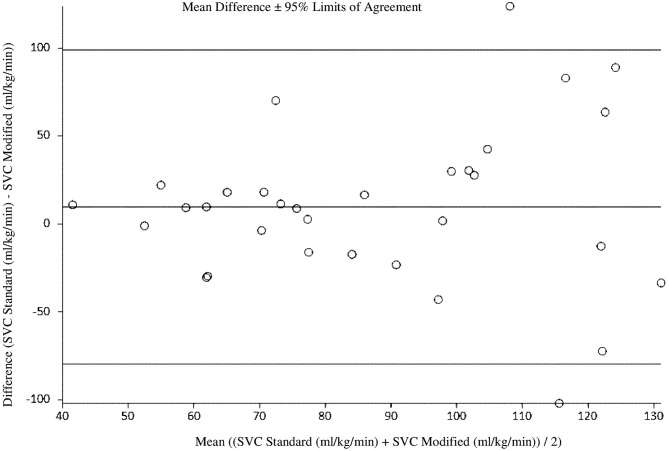
Agreement between standard and modified Superior Vena Cava (SVC) flow measurement at 18 h of age with mean difference and 95% limits of agreement.

At 18 h of age, the standard SVC flow method did not significantly correlate with the modified method (*r* = 0.09, *p* = 0.64). The mean difference (bias) between the measurements was +9.5 mL/kg/min, with 95% limits of agreement −79.6 to 98.7 mL/kg/min (Figure 2, Tables 3, 4). Heart rate correlated strongly and significantly between both methods at this time point. Area of the SVC had statistically significant correlation at 18 h of age. The VTI did not correlate at this time point (Table 3). The mean of differences, median of differences and 95% limits of agreement for the area of the SVC, VTI and heart rate for 18 h of age are included in Table 4.

**Figure 3:**
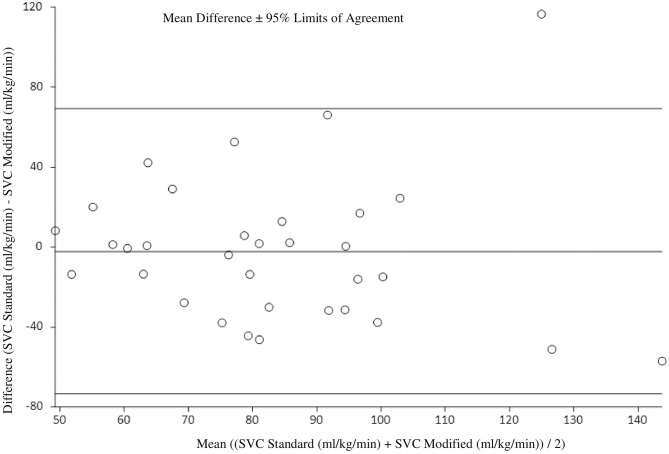
Agreement between standard and modified Superior Vena Cava (SVC) flow measurement at 36 h of age with mean difference and 95% limits of agreement.

At 36 h of age, the standard SVC flow method did not significantly correlate with the modified method (*r* = 0.17, *p* = 0.35). The mean difference (bias) between the measurements was −2.2 mL/kg/min with 95% limits of agreement −73.4 to 69.1 mL/kg/min (Figure 3, Tables 3, 4). Heart rate correlated strongly and significantly between both methods at this time point. Area of the SVC and VTI did not correlate at this time point (Table 3). The mean of differences, median of differences and 95% limits of agreement for the area of the SVC, VTI and heart rate for 36 h of age are included in Table 4.

## Discussion

The standard and modified methods have yielded clinically equivalent results at 6 and 36 h of age as defined in the design of the study. There was no statistically significant difference between the mean SVC flow for the cohort measured by either technique at any of the three time points. However, the SVC flow measurements correlated significantly only at 6 h of age. Interestingly, the mean differences (bias) between the two techniques were much smaller in our study (−0.8, 9.5, and −2.2 mL/kg/min, respectively) compared to only other report comparing these two techniques, 19 mL/kg/min in the study by Ficial et al. (26).

Despite clinically equivalent results, the agreement between the two methods was not satisfactory in our opinion, with very wide agreement limits at all three time points and as such we would not deem the two methods interchangeable.

Not surprisingly there was a strong, statistically significant correlation between the heart rate data at all time points as both SVC measurement techniques were done in immediate succession and the heart rate measurements are not related to the echocardiography technique.

SVC area correlated strongly and statistically significantly between the methods at 6 h of age. This correlation weakened with time, however was still statistically significant at 18 h of age. We would speculate from our own experience and the experience of others that the measurement of SVC flow and namely the parasternal diameter of the vessel, is more difficult to carry out and less accurate over time after delivery due to expanding lung fields (28). SVC area assessed by standard technique obtained consistently lower values compared with the modified technique with mean difference ranging from 1.8 to 2.9 mm^2^. This finding would be in agreement with finding of Ficial et al., albeit their mean difference was somewhat higher (4 mm^2^). When SVC area by echocardiography measurement was tested against phase contrast cardiac MRI measurement, both echocardiography techniques underestimated MRI data (24, 26). We would agree with speculation that MRI area measurement is likely to be superior in accurately obtaining the cross sectional area of the vessel (23). As highlighted above, there was a reasonable correlation between the two echocardiography methods in SVC cross sectional area estimation, and suprasternal access might be preferable as it is most likely better reflecting the true cross sectional area and seems easier to obtain after 24–48 h of age.

SVC VTI correlated statistically significantly between both techniques only at 6 h of age and the correlation weakened with time. The standard method consistently produced higher values compared with the modified technique, and the mean difference was very similar across the three time points (3.1, 3.0, 3.1 cm, respectively). This would be similar to mean difference in VTI's as previously reported (4.3 cm and 0.67 cm by Ficial et al. and Harabor et al., respectively) (25, 26). We would speculate that the lower difference published by Harabor is a result of very frequent angle correction use in their study. We predefined criteria for the use of angle correction (to use only when the angle between the SVC at the point of measure and the ultrasound beam was >15°) and we were successful in obtaining images without any need for this correction. Ficial et al. did not allow for angle correction in their study, thus their results are comparable with and similar to ours. In contrast to cross sectional area measurement, where MRI seems to be superior, we believe that in preterm infants with higher heart rates, MRI significantly underestimates true maximum velocity secondary to the relative low frame rate (20 images per cardiac cycle) compared with the current generation echocardiography machines (commonly >50 images per cardiac cycle) (23, 29). Thus the standard echocardiography technique would be most likely to reflect true VTI.

Our study is a first report of head to head comparison of two techniques of SVC flow measurements in the main population of interest, extremely preterm infants immediately after delivery. All paired measurements were done by the same investigator to eliminate inter-observer variability.

There are some limitations to our observations. Firstly our cohort of infants was relatively small (39 infants), albeit this represents substantial cohort of the most vulnerable preterm infants. We did not calculate intra-observer variability for this study. However, all measurements were done by operators with vast experience in functional neonatal echocardiography and SVC measurements. Also, as there is a lack of gold standard, we can only compare variabilities of the two techniques without comparing them to a gold standard.

In summary, both SVC flow echocardiography measurement techniques yielded clinically equivalent results, although due to poor correlation and agreement they do not seem to be interchangeable. The poor correlation is mostly secondary to the VTI measurements and we would strongly advocate use of the standard technique. The SVC cross-sectional area had quite satisfactory correlation early after delivery and in fact it seems plausible that the modified technique obtains more stable and accurate values. We would recommend that future studies use modified cross sectional area measurement together with standard SVC VTI measurements and correlate these with clinically relevant outcomes, and indeed with any future gold standard cardiac output measurement, in extremely premature babies in the transitional period after birth.

## Data Availability Statement

The raw data supporting the conclusions of this article will be made available by the authors, without undue reservation.

## Ethics Statement

The studies involving human participants were reviewed and approved by Research Ethics Committee in the Coombe Women and Infants University Hospital (No 23 - 2015). Written informed consent to participate in this study was provided by the participants' legal guardian/next of kin.

## Author Contributions

JM conceptualized and designed the study and contributed substantially to enrolment, measurements and data collection. He contributed substantially to data analysis and drafted the initial and final version of the manuscript. ZS was involved in the design of the study, contributed to data analysis and reviewed and revised the manuscript. NÓC contributed substantially to the enrolment to the study and reviewed and revised the manuscript. JJ contributed to design of the study and data analysis and reviewed and revised the manuscript. JS contributed substantially to design, enrolment, measurements in the study and data collection. She reviewed and revised the manuscript critically for important intellectual content. All the authors approved the final manuscript as submitted. They agree to be accountable for all aspects of the work.

## Conflict of Interest

The authors declare that the research was conducted in the absence of any commercial or financial relationships that could be construed as a potential conflict of interest.
